# Altered Subpopulations of Red Blood Cells and Post-treatment Anemia in Malaria

**DOI:** 10.3389/fphys.2022.875189

**Published:** 2022-04-05

**Authors:** Charlotte Chambrion, Mallorie Depond, Lucia Angella, Oussama Mouri, Eric Kendjo, Aurélie Fricot-Monsinjon, Camille Roussel, Sylvestre Biligui, Ilhame Tantaoui, Aida Taieb, Nicolas Argy, Sandrine Houzé, Renaud Piarroux, Jean-Yves Siriez, Stéphane Jaureguiberry, Sébastien Larréché, Marc Théllier, Nicolas Cenac, Pierre Buffet, Papa Alioune Ndour

**Affiliations:** ^1^ Biologie Intégrée du Globule Rouge, Université de Paris, Université des Antilles, Paris, France; ^2^ Centre National de Référence du Paludisme, Hôpital Pitié Salpêtrière, Paris, France; ^3^ Laboratoire d'Hématologie, Hôpital Universitaire Necker-Enfants Malades, Paris, France; ^4^ Centre National de Référence du Paludisme, Laboratoire de Parasitologie-Mycologie, Hôpital Bichat-Claude Bernard, Paris, France; ^5^ Sorbonne Université, Centre National de Référence du Paludisme, Hôpital Pitié-Salpêtriére, Paris, France; ^6^ Service d'accueil des Urgences Pédiatriques, Hôpital Robert Debré, Paris, France; ^7^ Service Maladies Infectieuses et Tropicales, Hôpital Bicêtre, Krimlin Bicêtre, France; ^8^ Département de Biologie Médicale, Hôpital d’Instruction Des Armées Bégin, Saint-Mandé, France; ^9^ IRSD, INSERM, INRA, INPENVT, Université de Toulouse, Toulouse, France

**Keywords:** P. falcipaprum, artemisinin derivatives, malaria anemia, RBC deformability, spleen filtering funcion, pitted or once infected RBC, membrane lipid balance

## Abstract

In acute malaria, the bulk of erythrocyte loss occurs after therapy, with a nadir of hemoglobin generally observed 3–7 days after treatment. The fine mechanisms leading to this early post-treatment anemia are still elusive. We explored pathological changes in RBC subpopulations by quantifying biochemical and mechanical alterations during severe malaria treated with artemisinin derivatives, a drug family that induce “pitting” in the spleen. In this study, the hemoglobin concentration dropped by 1.93 G/dl during therapy. During the same period, iRBC accounting for 6.12% of all RBC before therapy (BT) were replaced by pitted-RBC, accounting for 5.33% of RBC after therapy (AT). RBC loss was thus of 15.9%, of which only a minor part was due to the loss of iRBC or pitted-RBC. When comparing RBC BT and AT to normal controls, lipidomics revealed an increase in the cholesterol/phosphatidylethanolamine ratio (0.17 versus 0.24, *p* < 0.001) and cholesterol/phosphatidylinositol ratio (0.36 versus 0.67, *p* = 0.001). Using ektacytometry, we observed a reduced deformability of circulating RBC, similar BT and AT, compared to health control donors. The mean Elongation Index at 1.69Pa was 0.24 BT and 0.23 AT vs. 0.28 in controls (*p* < 0.0001). At 30Pa EI was 0.56 BT and 0.56 AT vs. 0.60 in controls (*p* < 0.001). The retention rate (rr) of RBC subpopulations in spleen-mimetic microsphere layers was higher for iRBC (rr = 20% *p* = 0.0033) and pitted-RBC (rr = 19%, *p* = 0.0031) than for healthy RBC (0.12%). Somewhat surprisingly, the post-treatment anemia in malaria results from the elimination of RBC that were never infected.

## Introduction

Malaria is the deadliest parasitic disease worldwide with half of the population exposed to the risk of infection by *Plasmodium falciparum*. The leading cause for morbidity and mortality in malaria is anemia, mainly due to the loss of red blood cells (RBC), that predominantly occurs after treatment (Price et al., 2001; Jaureguiberry et al., 2014; White 2018). Multiple RBC alterations have been described during malaria, including decreased deformability (Cranston et al., 1984; Safeukui et al., 2013; Jaureguiberry et al., 2014) that correlates with disease severity (Dondorp et al., 1999; Dondorp et al., 2002). These alterations may contribute to RBC clearance in the spleen. Less deformable RBC are indeed mechanically retained in inter-endothelial slits in the splenic red pulp (Deplaine et al., 2011). The lipid composition of the RBC membrane also interferes with the ability of RBC to stay in circulation. By interacting with spectrin, plasma membrane lipid content plays indeed an important role in the deformability and stability of RBC. Alterations such as the increase in the cholesterol/phospholipid ratio lead to a decrease in membrane fluidity and also compromise the osmotic balance (Sagawa and Shiraki 1978; Chabanel et al., 1983; Sarkar et al., 2018; Martinez-Vieyra et al., 2019). Multiple surface alterations of infected and uninfected RBC have also been described (Leech et al., 1984; Pouvelle et al., 2000; Awah et al., 2011).

The exploration of how malaria induces anemia is further complexified by the emergence of several RBC subpopulations before and after therapy. Infection obviously creates a new subpopulation of infected RBC (iRBC) with increasing molecular alterations as the parasite matures in its host cell (Fraser et al., 2021), but therapy also induces the emergence of new subsets. Artemisinin derivatives (AD), the most effective antimalarial drugs available, recommended as first-line treatment for severe malaria (WHO Report, 2010), act on all asexual parasite stages, including circulating rings (Rolling et al., Udomsangpetch et al., 1996, Dondorp et al., 2005) When RBC containing AD-altered rings cross the spleen, the parasite remnant is expelled and the pitted RBC returns to the circulation (Angus et al., 1997). The relative contributions to malarial anemia of the clearance of iRBC, pitted RBC and RBC that were never infected (niRBC) have not been analyzed in detail so far in early post-treatment anemia.

We explored the modifications of biochemical and functional properties of circulating RBC in patients with severe malaria before and after treatment with AD, with focus on iRBC and pitted RBC.

## Materials and Methods

### Study Cohort

Between 2017 and 2021, blood samples were collected from 29 patients with imported severe malaria with hyperparasitemia (>4% parasitemia) in France. Severe cases were defined by a positive blood smear with asexual forms of *P*. *falciparum* associated with at least one of the WHO’s severity criteria. All the patients included were involved in the national surveillance program of the French National Reference Center for Malaria and were treated by first-line treatment with intravenous artesunate (2.4 mg/kg, Guilin Pharmaceutical), or other artemisinin derivatives (Eurartesim 320 mg/40 mg, ≥ 35 kg: four tablets; or artemether Lumefantrine 20 mg/120 mg, ≥ 75 kg: 4 tablets).

### Lipidomic Analysis by HPLC and MS/MS

RBC from patients before or after treatment and RBC control from blood bank were washed 4 times in RPMI 1640 GlutaMAX containing HEPES (Life Technologies) and cryopreserved in 50% PBS1X in liquid nitrogen until lipidomic analysis by HPLC and MS/MS. Lipid extraction was performed according to a modified chloroform/methanol (2:1, v/v) procedure (Petkovic et al., 2005). Briefly, frozen samples were transferred into a 15 ml tube and 2 ml of chloroform/methanol (2:1, v/v) was added; chloroform (Sigma 288,,306-1 L), methanol (Fisher 10675112). The suspension was vortexed and incubated at room temperature with agitation for 20 min. After the incubation, 400 µL of 0.9% NaCl was added to the mixture and was then vortexed and centrifuged at 500 x g for 10 min to separate the organic phase from the aqueous phase. The chloroform (lower) layer was transferred into a new 1.5 ml tube and was centrifuged at 500 x g for 10 min. Finally, the supernatant was collected without touching the pellet and was transferred into a new 1.5 ml tube. Internal standards (Cer d18:1/15:0, 16 ng; PE 12:0/12:0, 180 ng; PC 13:0/13:0, 16 ng; SM d18:1/12:0, 16 ng; PI 16:0/17:0, 30 ng; PS 12:0/12:0, 156.25 ng; all from Avanti Polar Lipids) were added to 45 µL of lipid extract. Sample solutions were analyzed using an Agilent 1290 UPLC system coupled to a G6460 triple quadripole spectrometer (Agilent Technologies). MassHunter software was used for data acquisition and analysis. A Kinetex HILIC column (Phenomenex, 50 × 4.6 mm, 2.6 μm) was used for LC separations. The column temperature was maintained at 40°C. Mobile phase A was acetonitrile and B was 10 mM ammonium formate in water at pH 3.2. The gradient was as follows: from 10 to 30% B in 10 min, 100% B from 10 to 12 min, and then back to 10% B at 13 min for 1 min to re-equilibrate prior to the next injection. The flow rate of the mobile phase was 0.3 ml/min, and the injection volume was 5 μL. An electrospray source was employed in positive (for Cer, PE, PC, and SM analysis) or negative ion mode (for PI and PS analysis). The collision gas was nitrogen. Needle voltage was set at +4,000 V. Several scan modes were used. First, to obtain the naturally different masses of different species, we analyzed cell lipid extracts with a precursor ion scan at 184 m/z, 241 m/z, and 264 m/z for PC/SM, PI, and Cer, respectively. We performed a neutral loss scan at 141 and 87 m/z for PE and PS, respectively. The collision energy optimums for Cer, PE, PC, SM, PI, and PS were 25 eV, 20 eV, 30 eV, 25 eV, 45 eV, and 22 eV, respectively. The corresponding SRM transitions were used to quantify different phospholipid species for each class. Two MRM acquisitions were necessary, due to important differences between phospholipid classes. Data were treated with QqQ Quantitative (vB.05.00) and Qualitative analysis software (vB.04.00).

### Parasitized and Pitted RBC Staining

RBC from patients before or after treatment and RBC control were washed 4 times in RPMI 1640 GlutaMAX containing HEPES (Life Technologies) and fixed with 1% glutaraldehyde diluted in PBS 1X. After two washes in PBS1X, samples were incubated with a human polyclonal hyper immune serum (Buffet et al., 2006) at 1:10 in PBS with 1% Albumax II (Life Technologies) and 0.1% Triton X100 (Sigma Aldrich) for membrane permeabilization. After 25 min of incubation under agitation, samples were washed two times in PBS1X and incubated with a secondary antibody goat anti-human IgG coupled with either Alexa Fluor 488 or 568 at 1:500 (Life technologies) or Dylight755 (Invitrogen) at 1:100. Based on the color of the secondary antibody already used, DNA was labeled with either Hoechst 34,580 at 1:1000 or SybrGreen at 1:5000 (Life Technologies) for 25 min. Parasitemia and pittemia were finally measured by the cytometer BD FACSCanto^TM^II, BD Biosciences and analyses were performed using FlowJo software.

### Deformability Assessment by Ektacytometry

After blood collection, elongation indexes of each sample diluted at 1:100 in Polyvinylpyrrolidone polymers buffer (RR mechatronics) were measured by ektacytometry with shear stress from 0.3 to 30Pa. All the samples were processed within 48 h after blood collection. A laser-assisted optical rotational cell analyzer (LORRCA, RR Mechatronics) was used to calculate the elongation index, which is defined as the ratio of the difference between two axes of the ellipsoidal diffraction pattern and the sum of these two axes. An elongation index at 1.69 Pa shear stress reflects deformability due to membrane rigidity and 30 Pa shear stress reflects deformability due to intern viscosity (Baskurt OK et al. Clin Hemorheol Microcirc. 2009). A one-way Anova parametric unpaired t-test with Bonferroni’s correction was performed for statistical analysis.

### Microsphiltration Assays

Filtration of RBCs was performed using a mix of calibrated metal microbeads (Industrie des Poudres Sphériques, France), composed of 70% of 5–15 µm diameter and 30% of 15–25 µm diameter and supported by a layer of 25–45 µm diameter microbeads. The beads were distributed in a special 96-well plate adapted for microsphiltration as described in Deplaine et al. Blood 2011. RBCs were prepared at 2% hematocrit (or 1% if parasitemia exceeded 10%) in PBS 1X/1% Albumax II. For each well, 200 µL of RBC suspension were loaded and filtered by aspiration. Wells were flushed with 1.6 ml of PBS/1% Albumax II and downstream suspension was collected. Healthy RBC fixed with 1% glutaraldehyde or not were used as controls. Retention rate was calculated as following [(% Downstream - % Upstream)/% Upstream] x100. Positive values represented enrichments and negative values retentions.

### Statistics

Statistical analyses were performed using GraphPad PRISM six software. For Hb comparison before and after therapy, only patients with available data in both periods were analyzed by a paired t-test with a normal distribution (D'Agostino and Pearson test). Differences at *p < 0.05* were considered significant. For populational analysis, deformability index and retention rate in microsphiltration, RBC parameters were compared before and after therapy and with healthy RBC from blood bank. Anova test was used when appropriate with a Bonferroni correction. One asterisk (*) identifies adjusted *p* values between 0.01 and 0.05, two asterisks (**) identify adjusted *p* values between 0.01 and 0.001.

## Results

### Magnitude of Early Post-treatment Anemia and Clearance of Different RBC Subpopulations

**FIGURE 1:**
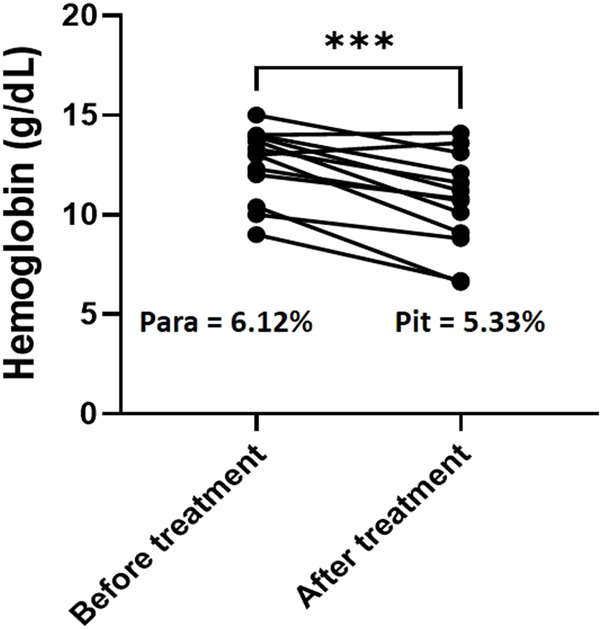
Hemoglobin decline and RBC clearance. Hemoglobin concentration for the 13 patients with Hb data available both before and after therapy. Paired t-test, *p* value for hemoglobin comparison = 0.0003. Para: mean parasitemia for the same patients before treatment with AD.; pit: mean pittemia for the same patient after treatment with AD.

Hemoglobin (Hb) decline was 1.93G/dl with a mean Hb level of BT of 12.86 G/dl vs. 11.01 G/dl AT (*n* = 13, *p* = 0.0003, Figure 1). RBC loss was thus of 15.9% (or less since a small part of the hemoglobin drop was due to hemodilution AT (Levin et al., 2001; Drevon et al., 2021). During the same period, the loss of iRBC was 5.9%, and the gain of pitted RBC was 5.1% of all RBC, corresponding to a net loss of 0.8% of total RBC. Altogether, the net loss of the subpopulation of RBC directly modified by invasion of RBC by parasites accounted for minor part of the total loss of RBC during this period. The bulk of Hb loss was therefore related to the elimination of RBC that had never been infected (niRBC).

### Infection and Treatment Alter the Cholesterol/Phospholipids Ratio in the Membrane of Circulating RBC

**FIGURE 2:**
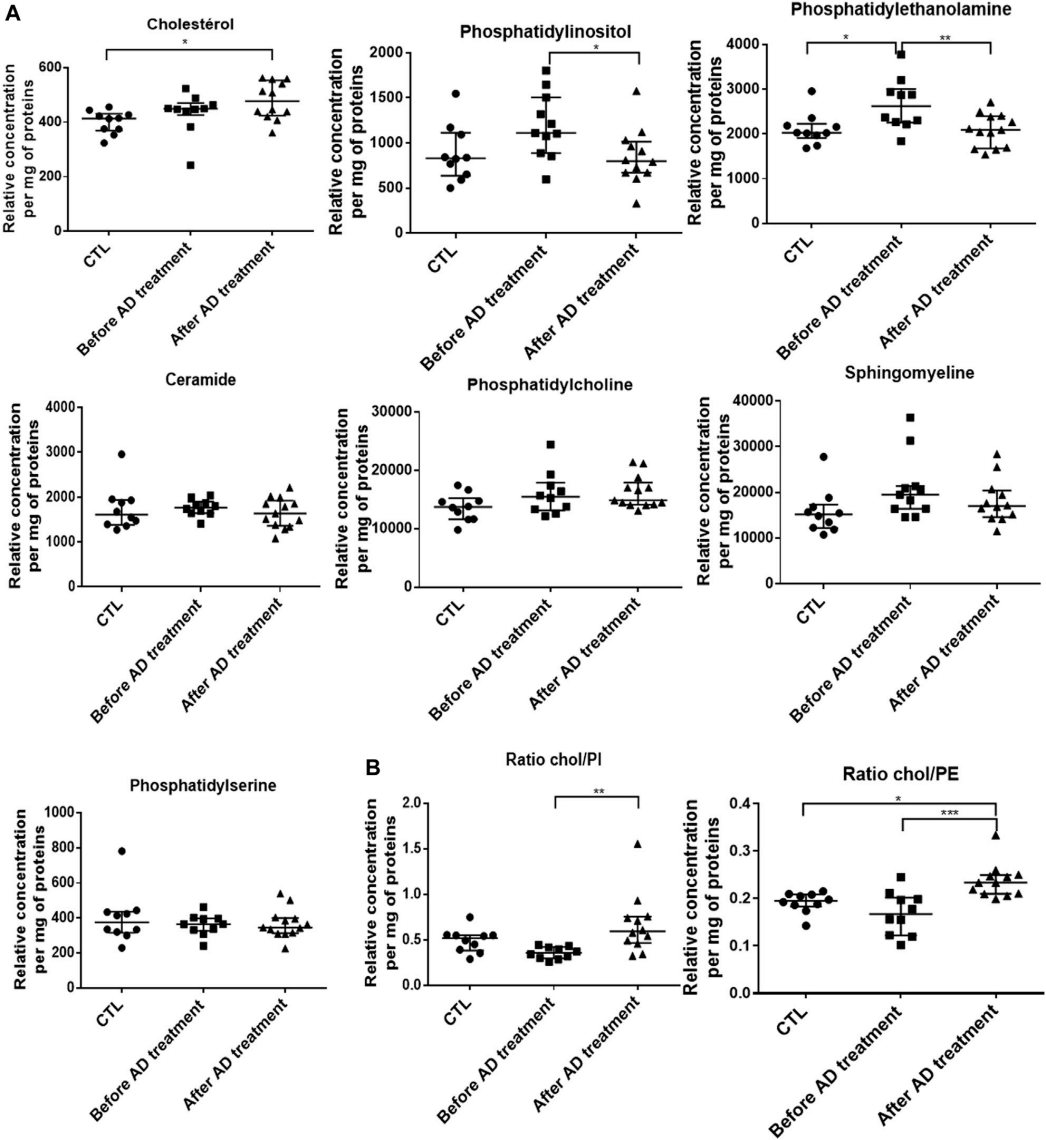
Alterations of cholesterol and phospholipid RBC membrane composition before and after antimalarial therapy in severe malaria. **(A)**. Lipid content of RBC membranes using HPLC and MS/MS on samples from 10 healthy donors (CTL), patients sampled before (N = 10) and after (N = 12) antimalarial therapy. **(B)**. Cholesterol/Phosphatidylethanolamine (ratio Chol/PE) and Cholesterol/Phosphatidylinositol (ratio Chol/PI). Stars indicate significant differences.

We measured the composition of lipids (*de novo* biosynthesis or uptake by *P. falciparum*) in RBC samples BT and at complete clearance of parasites AT. A lipidomic analysis including cholesterol (Chol), four phospholipids (phosphatidylethanolamine: PE, phosphatidylinositol: PI, phosphatidylcholine: PC and phosphatidylserine: PS), and two sphingolipids (ceramide and sphingomyelin) was performed by HPLC and MS/MS on membrane extracts from RBC collected in infected patients BT and AT. RBC from healthy donors were used as controls. Regarding ceramide, PC, PS and sphingomyelin, no significant variations were observed between RBC from healthy donors and RBC from infected patients either BT or AT. Conversely, compared to healthy controls (400.5 arbitrary units (AU), *n* = 12, Figure 2), total Chol amounts were slightly higher than controls BT (433.6, *p* = 0.491) and significantly higher than controls AT (479.5 AU, *p* = 0.020). Compared to healthy controls, PE and PI contents were higher BT (2,111 vs. 2665 AU, *n* = 10, *p* = 0.021 for PE and 883 vs. 1192 AU, *n* = 10, *p* = 0.095 for PI, Figure 2A); and then decreased significantly AT to control levels (2082 AU, *n* = 12, *p* = 0.009 for PE and 850 AU, n = 12, *p* = 0.0462 for PI, Figure 2A). When we analyzed Chol/PE or Chol/PI ratio that are associated with membrane deformability and stability, both the Chol/PI and Chol/PE ratio increased significantly AT compared to BT (0.24 vs. 0.17, *p* < 0.001 for Chol/PE and 0.67 vs. 0.36 for Chol/PI, *p* = 0.001, Figure 2B). These results suggest that infection, treatment, and pitting altered the lipid composition of RBC plasma membranes.

### The Deformability and Retention of Circulating RBC Is Similarly Reduced Before and After Treatment

**FIGURE 3:**
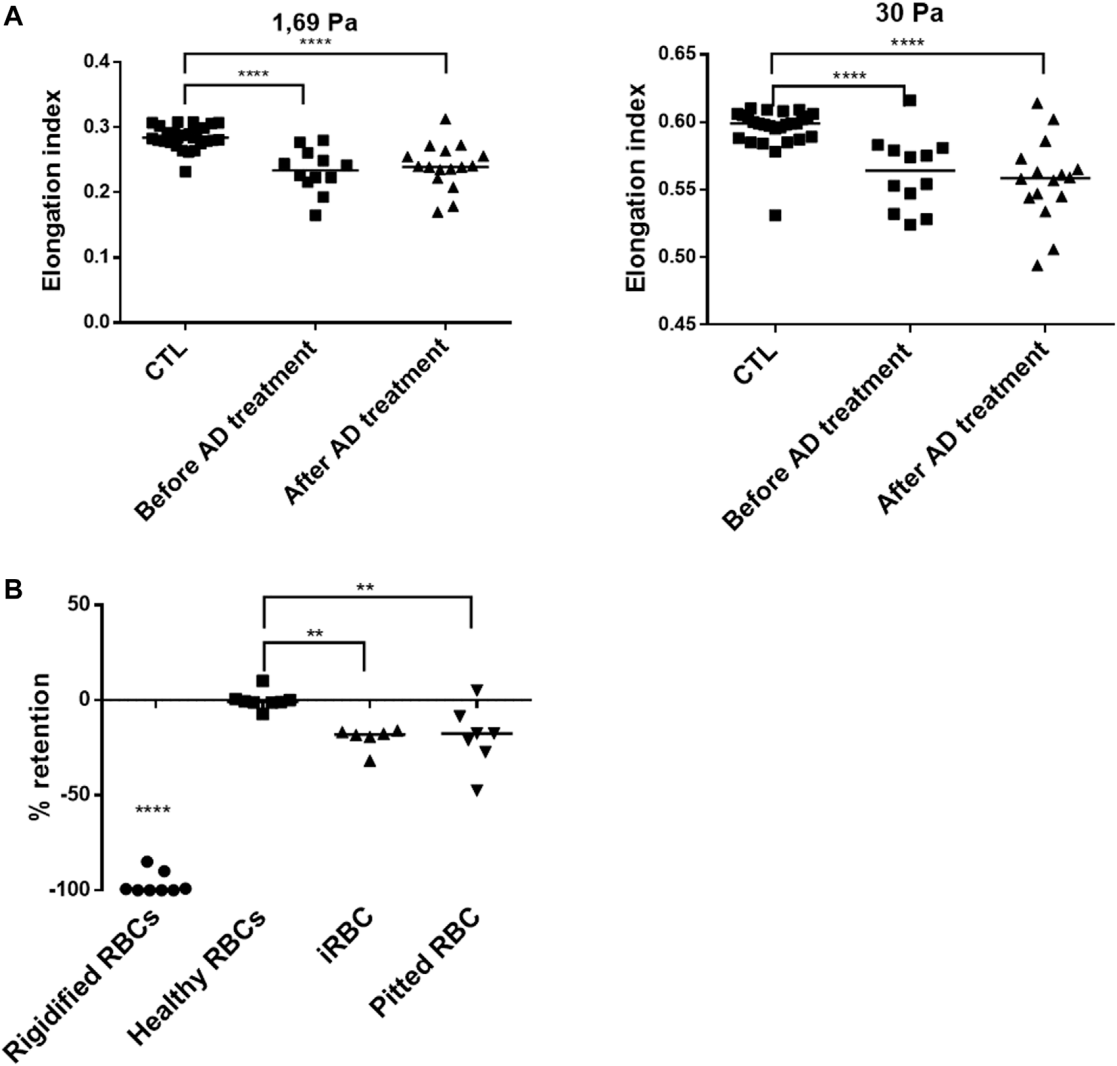
Altered functional properties of circulating RBCs. **(A)**. Elongation index at different shear stresses of RBC from patients before antimalarial therapy (*n* = 12) or upon follow-up (*n* = 16), compared to RBCs from healthy donors (CTL, *n* = 27). **(B)**. Retention of glutaraldehyde-fixed RBC (Rigidified RBC, *n* = 8), healthy RBC (*n* = 8), patient iRBC (*n* = 6), and pitted RBC (*n* = 7). Negative values reflect retention in microspheres; positive values reflect enrichment.

Changes in the lipid contents of RBCs have been shown to be associated to altered deformability and may contribute to their elimination by the spleen (Sarkar et al., 2018; Martinez-Vieyra et al., 2019). The deformability of RBC collected either BT (parasitemia ranging from 1 to 10.7%, *n* = 12) or AT (concentrations of pitted RBC ranging from 0.4 to 12%, *n* = 16), was quantified by ektacytometry and compared to RBC from healthy donors (CTL, *n* = 27). RBC deformability was significantly lower than controls both BT and AT with and elongation Index (EI) at 1.69 Pa of 0.24 BT and 0.23 AT vs. 0.28 in healthy donors (*n* = 28, *p* < 0.0001). At 30 Pa EI was 0.56 BT and 0.56 AT vs. 0.60 in healthy donors (*n* = 27, *p* < 0.001 (Figures 3A, B). This suggests that their decreased deformability is related to infection-related increase in membrane rigidity or internal viscosity, or both. These results also show that alterations in circulating RBC are not immediately restored after treatment with AD. Finally, the ability of RBC subpopulations (iRBC BT and pitted RBC AT) to cross splenic inter-endothelial slits was assessed using a spleen-mimetic method where microsphere-based filters quantify the retention rates (rr) of RBC subpopulations (Deplaine et al., 2011; Ndour et al., 2015; Depond et al., 2019) (Figure 3B). Both iRBC (*n* = 6) and pitted RBC (*n* = 7) were more retained in microsphere layers than healthy controls (*n* = 8), at strikingly similar levels (mean rr iRBC: 20% and rr pitted RBC: 19%, Figure 3C).

## Discussion

Our careful analysis of RBC properties in patients treated by AD for severe malaria shows that RBC from treated patients display substantially impaired functional alterations. iRBC and pitted RBC, the two subpopulations directly altered by parasite invasion followed by pitting, display similarly altered properties. The intensity of these alterations should result in their clearance by a normal spleen, but surprisingly they are observed in circulation. This suggest that the spleen less stringently senses and retains altered RBC during severe malaria than at baseline. Early anemia occurs despite this relative hyposplenism, and in this cohort, the bulk of Hb loss was therefore likely related to the elimination of RBC that had never been infected (niRBC). The fine mechanisms or their “by-stander” alteration and early clearance remain to be determined.

To maintain tissue homeostasis, healthy red blood cells (RBC) circulate in the vessels and non-vascularized areas in a permanent flow. After infection, *P. falciparum* acts to restore a nucleus in mature RBCs with *de novo* transcription, protein synthesis, and trafficking. Here, we explored the changes caused by the infection in different RBC subpopulations and their subsequent clearance following treatment of patients with the artemisinin derivatives (AD), the first-line recommended antimalarial therapy. Upon entry of merozoite, the properties of RBC plasma membrane complex are extensively modified through its association with early expressed parasite proteins such as the Ring-infected erythrocyte surface antigen (RESA), which increase stability and rigidity (Pei et al., 2007).

In the context of malaria treated with AD, this maturation of circulating ring-infected RBC is rapidly stopped, and dead parasites are expelled from the host RBC without lysis in the pitting process (Chotivanich et al., 2000; Buffet et al., 2006; Ndour et al., 2015), the major mechanism of parasite clearance in malaria treated with AD (Ndour et al., 2015). In this study, ektacytometry—which measures a populational deformability index (Cranston et al., 1984)—showed a general decrease of this deformability in circulating RBC during infection and after treatment. This decreased deformability is then associated with anemia through a reduced hemoglobin (Hb) concentration (-1.93 g/dl) in patients treated with AD. By exploring the ability of the different RBC subpopulations to cross the spleen using microsphiltration assays before and after treatment, we showed that iRBC and pitted RBC displayed a similar increased retention rate in clinical samples. This likely reflects similar alterations that result in the further elimination at least of pitted RBC during delayed anemia (Jaureguiberry et al., 2014), since the early drop in Hb (-15.9%) observed here is not explained by the decrease of iRBC and pitted RBC (-0.8% of all RBC during the three to five first day post-infection and therapy). We could not exclude that the dynamics and physiological mechanism of the infection of RBC, pitting and subsequent elimination of those subpopulations, can be hidden in the circulating RBC by the kinetics of the spleen filtering function. However, our results suggest that altered never infected (ni)RBC explain presumably the remaining biomass of eliminated RBC. In fact, in addition to parasite proteins, other factors may impact and deteriorate deformability and the ability of RBC to circulate, since the content and balance of plasma membrane lipids may also change the biomechanical characteristics of RBC (Chabanel et al., 1983; Janero 1990; Martinez-Vieyra et al., 2019) and this balance is also dependent of RBC environment, where certain lipids directly come from, so infection environment is key determinant. Phospholipids such as phosphatidylserine (PS), phosphatidylethanolamine (PE), and phosphatidylinositol (PI) interact also with spectrin, contributing to the deformability and stability of erythrocytes by maintaining membrane asymmetry (Sarkar et al., 2018). *P. falciparum* is able to produce *de novo* several lipids such as phosphatidylcholine (PC), essential for its metabolism (Pessi et al., 2004), but it lacks a *de novo* cholesterol (Chol) biosynthesis pathway. Parasites need to uptake it from its immediate blood environment via the RBC plasma membrane (Hayakawa et al., 2021). Although Chol has been described to be depleted from the blood and surface membrane of iRBCs during maturation (Tran et al., 2016; Fraser et al., 2021; Hayakawa et al., 2021), we observe a general increase of Chol in whole cell analysis of samples containing iRBC and then pitted RBC following treatment with AD. This likely reflects the sequestered Chol in circulating iRBC replaced later by pitted RBC or induced changes in the blood flow lipid composition affecting niRBC. While most of the other lipids studied did not vary during infection and after treatment or slightly increased (PC), lipidomic analysis revealed that several lipids from parasite *de novo* biosynthesis (PE and PI) increased during infection in samples containing iRBC and then decreased significantly after treatment and removal of dead parasites. These complex variations reveal the balance between lipid uptake, transport, or biosynthesis by parasite, before metabolism being arrested by AD treatment. As a consequence of these lipid balance fluctuations and alterations after infection and treatment, we observed a general increase in the Chol/PI or PE ratio (Figure 2) associated with membrane destabilization (Chabanel et al., 1983; Janero 1990; Martinez-Vieyra et al., 2019).

Taken together, these data showed that infection by *P. falciparum* induces modification of the proteins and the lipids located in the RBC plasma membrane and cytosol, resulting in a reduced deformability associated with spleen retention and clearance from the blood circulation. These modifications are maintained after treatment with AD. These results also demonstrate that, in addition to specifically and profoundly affecting certain RBC subpopulations, infection and treatment with AD seem to largely alter the circulating niRBC populations. This may explain why the magnitude of niRBCs lost is often higher than iRBC in malaria anemia (Day et al., 1996; Price et al., 2001; White 2018). A similar mechanism was suggested with the loss of pitted RBC in post-artesunate delayed hemolysis where loss of pitted RBC is higher (30-to-70% of all RBC eliminated (Jaureguiberry et al., 2014)). The changes in the RBC property impact their ability to circulate, to cross the spleen and may also induce RBC lysis or elimination through complementary immune mechanisms (Fernandez-Arias et al., 2016; Fraser et al., 2021).

Conclusion: *P. falciparum* infection, artesunate treatment, and pitting induce changes in the biochemical properties of circulating RBC and the early post treatment anemia is mainly due to the clearance of never infected RBC. Given the fact that circulating RBC are supposed to correspond to cells that are found to meet the standard quality control of the spleen (Crosby, et al., 1959), we can speculate that the significant increase in alterations observed in circulating RBC (pitted RBC) is due to a temporal change of the spleen threshold of retention and the further spleen function restoration likely delayed hemolytic anemia few days later (Jaureguiberry et al., 2014). The fine mechanisms or their “by-stander” alteration and early clearance remain to be determined.

## Data Availability

The raw data supporting the conclusion of this article will be made available by the authors, without undue reservation.
